# The Book World of Medicine and Science

**Published:** 1911-04-29

**Authors:** 


### The Book World of Medicine and Science.

Outlines of Zoology. By J. Arthur Thomson, M.A. Fifth Edition. (London : Henry Frowde and Hodder and Stoughton. Pp. 811, with 420 illustrations, bibliography, and index. Price 12s. 6d. net.)

The fifth edition of this excellent textbook has been enhanced by many new figures, and it is pleasing to notice that Professor Thomson is endeavouring as much as possible to do away with the stereotyped illustrations which are becoming so familiar and are in many cases so unsatisfactory. The bibliography is a good feature of the work, but we are surprised to see no mention under the title "History of Zoology" of Emil Radl's "Geschichte der Biologischen Theorien" (2 vols., Engelmann, Leipzig, 1909), which is undoubtedly the best book on the subject which has yet appeared. Professor Thomson deals in a clear and just manner with the controversial problems of biology, and, being the professed disciple of no school, states the theories with their supporting facts briefly and concisely. Under the title of "Books on Evolution" we would suggest the inclusion of Samuel Butler's "Unconscious Memory."

The Cookery Annual for 1911 and Year Book of the Universal Cookery and Food Association. Edited by C. Herman Senn. (The Food and Cookery Publishing Agency, Westminster, S.W. 1s. net.)

This little volume is an entertaining annual devoted more especially to the interests of the Universal Food and Cookery Association and its affiliated societies, such as the Réunion des Gastronomes, and the Benevolent Society. The Association exists for the promotion and encouragement of the art of cooking generally, and appears to be a very representative body, although we should like to see on its list of patrons the names of a few more dietarians of established reputation. A great number of very interesting menus—from a gastronomic as well as from a dietetic point of view—are given in this book, with a few recipes of less well-known dishes. The chapter on marketing hints at the end of the book, giving as it does some simple directions for detecting common adulterants in foodstuffs, is worth reading by everyone who has anything to do with their purchase.

The Local Government Annual and Official Directory for 1911. Edited by S. Edgecumbe Rogers. (The Local Government Journal Offices, 27a Farringdon Street, E.C. 1s. 6d. net.)

This is the "Whitaker" for the Local Government official, who will find in it a mass of information on all points in which he may be interested so long as these have any bearing on his work and the department with which he is concerned. To Medical Officers of Health it is an indispensable manual, and we commend it as well to members of Care Committees, to part-time officials in the service of the London County Council, and, in fact, to everybody who desires information on points in connection with the work of the Local Government Board. It is fully corrected and up to date, and therefore a trustworthy and thoroughly reliable little book of reference.

Memorias do Instituto Oswaldo Cruz. Ano 1910. Tomo II. Faciculo II. (Rio de Janeiro, Manguinhos. 1910. Pp. 144, with 13 illustrations.)

This volume contains eight special articles. Dr. S. Von Prowazek contributes an article on Brazilian protozoa; Drs. Max Hartmann and Carlos Chagas a study of nuclear division in the hyaline amœba Dang, the last-named worker an account of the cytology of a new coccidium, the *Adelea Hartmanni*, from the intestine of the *Dysdercus ruficollis*, L.; Dr. A. Fontes, a study of tuberculosis; Dr. Arthur Neiva, information on the biology of *Conorhinus megistus*, Burm.; Dr. Adolpho Lutz, a second contribution on Brazilian specius of the genus "Simulium"; Dr. Arthur Moses, on antidysenteric serum and its methods of administration, and Dr. Gomes de Faria a contribution towards the classification of Brazilian Entozoa, III. *Ankylostomum Braziliense*, n.sp. Parasite of cats and dogs. As formerly, each article is printed in Portuguese and another language, usually German. The last contribution is translated into English from the original Portuguese. Numerous illustrations from microphotographs and drawings by Mr. Castro Silva grace the volume, and once again we must congratulate the artist on the beauty of his work.

Report Originally Submitted to the Bucks County Council in the Year 1892. Containing letters from Miss Florence Nightingale on Health Visiting in Rural Districts. (London : P. S. King and Son. 1911. Pp. 61. Price 3d. net. Published for the National League for Physical Education and Improvement.)

This pamphlet has been reprinted in the hope that the movement for health visiting in rural districts may be stimulated and continued as a sort of memorial to the great nursing pioneer who recently passed to her reward. It is felt by the Society that it is by continuing and extending her life-work that Miss Nightingale would have wished her name to be perpetuated. The pamphlet contains correspondence that passed between her and Mr. Frederick W. Verney on the subject of "Health Visitors," as well as a syllabus of lectures given to health visitors, reports on their work, hints to them, a summary of the scheme, etc., the whole being of interest by reason of its marking the spread into the country of a scheme which was originally started in some of the larger towns, such as Manchester.

Man's Redemption of Man. By William Osler. (London : Constable and Co., Ltd. 1910. Price 1s. net.

This address was delivered at a service held for the students of the University of Edinburgh in connection with the meeting in that city of the National Association for the Prevention of Tuberculosis. Professor Osler points out what man has done for man in hygiene, preventive medicine, surgery, and anæsthesia.

The King's College Hospital Book of Cooking Recipes. (London : Longmans, Green and Co. 1911. Price 1s. net.)

This volume contains recipes contributed by professionals and amateurs. Some are said to be family "secrets" revealed for the first time, some are contributed by a Hindoo chef, and others are from ladies prominent in society. In case the title might lead one to suppose that the recipes are mainly intended for use in the preparation of food for the sick-room, it may be as well to mention at once that such is not the case. The book is intended for household use, and contains references to many things that, by reason of their comparative indigestibility, are by no means ideal dishes for the convalescent. Not that we mean to suggest that they are unsuitable. They happen merely to be intended for people in active life! The purchaser at the same time that she obtains value for her money will be helping a very deserving charity, and so we can doubly recommend the little volume.

